# Photoplethysmography Features Correlated with Blood Pressure Changes

**DOI:** 10.3390/diagnostics14202309

**Published:** 2024-10-17

**Authors:** Mohamed Elgendi, Elisabeth Jost, Aymen Alian, Richard Ribon Fletcher, Hagen Bomberg, Urs Eichenberger, Carlo Menon

**Affiliations:** 1 Department of Biomedical Engineering and Biotechnology, Khalifa University of Science and Technology, Abu Dhabi P.O. Box 127788, United Arab Emirates; 2 Healthcare Engineering Innovation Group (HEIG), Khalifa University of Science and Technology, Abu Dhabi P.O. Box 127788, United Arab Emirates; 3 Biomedical and Mobile Health Technology Research Lab, ETH Zürich, 8008 Zürich, Switzerland; ejost@student.ethz.ch; 4 Yale School of Medicine, Yale University, New Haven, CT 06510, USA; aymen.alian@yale.edu; 5 Department of Mechanical Engineering, Massachusetts Institute of Technology, Cambridge, MA 02139, USA; fletcher@media.mit.edu; 6 Department for Anesthesiology, Intensive Care and Pain Medicine, Balgrist University Hospital, 8008 Zürich, Switzerland; hagen.bomberg@balgrist.ch (H.B.); urs.eichenberger@balgrist.ch (U.E.)

**Keywords:** blood pressure estimation, PPG, vascular health, PPG features, systolic blood pressure prediction, second derivative of PPG, velocity photoplethysmogram, acceleration photoplethysmogram, temporal PPG features, wearable devices

## Abstract

Blood pressure measurement is a key indicator of vascular health and a routine part of medical examinations. Given the ability of photoplethysmography (PPG) signals to provide insights into the microvascular bed and their compatibility with wearable devices, significant research has focused on using PPG signals for blood pressure estimation. This study aimed to identify specific clinical PPG features that vary with different blood pressure levels. Through a literature review of 297 publications, we selected 16 relevant studies and identified key time-dependent PPG features associated with blood pressure prediction. Our analysis highlighted the second derivative of PPG signals, particularly the b/a and d/a ratios, as the most frequently reported and significant predictors of systolic blood pressure. Additionally, features from the velocity and acceleration photoplethysmograms were also notable. In total, 29 features were analyzed, revealing novel temporal domain features that show promise for further research and application in blood pressure estimation.

## 1. Introduction

Globally, about 1.28 billion adults between the ages of 30 and 79 suffer from hypertension [1]. Alarmingly, a majority of them, nearly two-thirds, reside in low- to middle-income countries [1]. Adding to the concern is the fact that approximately 46% of these individuals are unaware of their hypertensive condition. Due to the insidious nature of symptom development, blood pressure measurement is essential to observe hypertension [1].

Blood pressure is an important parameter that reflects vascular health and provides insights into cardiac function and the condition of the circulatory system. Abnormal blood pressure and variations in blood pressure levels are indicators of potential vascular health risks and play essential roles in the prevention and diagnosis of many cardiovascular diseases. Different blood pressure ranges define a spectrum of health conditions, each carrying specific associations with well-being, ranging from normotension, which reveals normal cardiovascular function, to hypertension and hypotension, which demand medical attention, indicating overall medical health [2]. Stroke and cardiovasular diseases are associated with hypertension and are the leading causes of death in the United States, with 691,095 deaths in 2021, whereas hypertension was a main cause or a co-factor [3]. Continuous and cuffless blood pressure monitoring surpasses the limitations of traditional blood pressure measurement. Cuffless-based methods enable the tracking of blood pressure over extended periods. By using PPG signals and advanced algorithms, especially machine learning, cuffless monitoring provides real-time insights into blood pressure trends and blood pressure ranges. Representative works in the domain include studies by Gupta et al. [4], Yao et al. [5], Ali et al. [6], El Hajj et al. [7], Chen et al. [8], and Maqsood et al. [9].

Photoplethysmography (PPG) signals are noninvasive, optical-based signals that provide information about the change in blood volume in a small vascular bed [10]. PPG waveforms are relatively easily acquired by placing a sensor on the skin of, for example, the fingertip, wrist, or earlobe. Furthermore, the device is lightweight and portable, which makes it easily integratable in portable devices such as smartwatches. PPG can be applied to a wide range of physiological parameters, including heart rate and vascular compliance. Its adaptability makes it a useful tool for assessing various aspects of cardiovascular health [11,12,13].

The relationship between PPG waveforms and blood pressure is still not fully understood, but various signal processing techniques such as time domain analysis, frequency domain analysis [14], and machine learning algorithms have been used to extract features from PPG waveforms and correlate them with blood pressure [15]. These studies have demonstrated promising results, suggesting the potential of estimating continuous blood pressure monitoring using PPG signals. However, the PPG features relevant to predict blood pressure remain unclear, as many features have been suggested, but the importance of specific features has not been evaluated. Two examples for such a case were given in the publications by Chowdhury et al. [14] and Duan et al. [15].

As described, the importance of finding characteristic features that could play a crucial role in promoting the classification of patients into specific blood pressure categories becomes apparent. In order to identify such features, we have conducted a review of a range of publications that analyzed the significance of diverse attributes within varying blood pressure classifications.

## 2. Methods

To comprehensively gather relevant scientific literature on the relationship between PPG signal features and blood pressure, a systematic search strategy was employed across multiple databases. The aim was to identify studies discussing time-dependent PPG features associated with blood pressure variation, such as hypertension and hypotension.

### 2.1. Search Strategy

We conducted searches in three major electronic databases: **PubMed**, **IEEE Xplore**, and **Embase**, and we supplemented these with manual searches from other sources. To ensure a comprehensive capture of relevant studies, we constructed three distinct search queries using logical operators (AND, OR) to target publications discussing PPG features in relation to blood pressure estimation.

The three search terms were as follows:

- **First search query:** *(Photoplethysmogram) AND (hypertension OR hypotension) AND (features OR second derivative)*.
- **Second search query:** *(Photoplethysmogram) AND (hypertension OR hypotension OR blood pressure) AND (SDPTG OR d/a ratio)*.
- **Third search query: ***(Photoplethysmogram OR photoplethysmography) AND (hypertension OR hypotension) AND (features OR derivative)*.

This approach was designed to maximize the capture of relevant research by covering a wide range of terminology and focusing on key aspects of PPG features used for blood pressure analysis.

### 2.2. Screening Process

After the search was conducted, we removed duplicate records and proceeded to screen the remaining articles based on their titles and abstracts. During this phase, we evaluated studies based on their relevance to the topic of PPG signal features and blood pressure estimation. The main criteria for this stage were the inclusion of time-dependent PPG features and a focus on hypertension or hypotension.

### 2.3. Full-Text Assessment

Studies that passed the initial screening were further assessed through full-text review to ensure they met all predefined eligibility criteria. This detailed review focused on:

- The inclusion of clinically relevant PPG signal features,
- Defined relationships between PPG features and blood pressure (e.g., systolic, diastolic),
- Appropriate methodologies related to feature extraction and analysis.

Studies that did not meet these criteria were excluded from further analysis. This step ensured that only high-quality, relevant research was included for the final evaluation.

### 2.4. Data Extraction

For the eligible studies, key information was extracted, focusing on the PPG features analyzed, the methods used for feature extraction, and the significance of the findings in relation to blood pressure estimation. The extracted data were then synthesized to provide a comprehensive overview of the current state of research on the use of PPG for blood pressure monitoring.

## 3. Results

**Figure 1:**
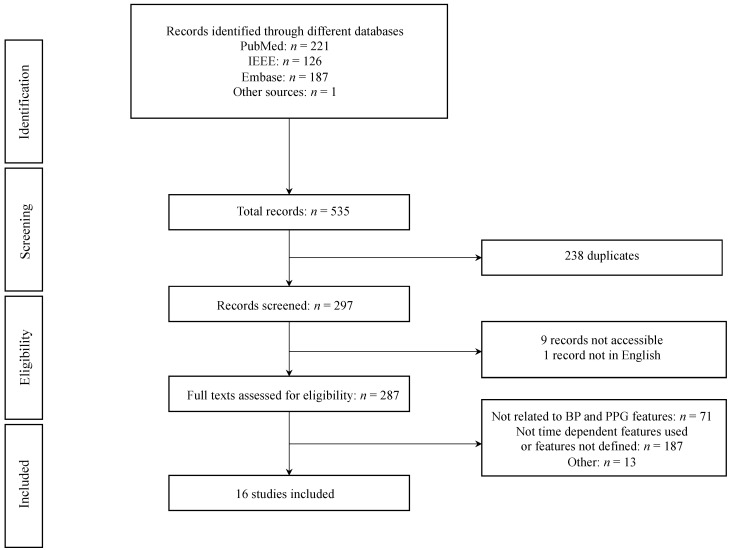
Workflow of the study. Identification, screening, eligibility, and inclusion of articles.

Following the implementation of the specified three search terms delineated within the methodology section, a total of 43 articles were identified on PubMed, 29 on IEEE, and 39 on Embase with the first search term. Subsequently, with the application of the second search term, we ascertained 19 research publications on PubMed, 1 on IEEE Xplore, and another 15 on Embase. Finally, the third search term yielded 159 papers from PubMed, 96 from IEEE Xplore, and 133 from Embase. During the review of relevant literature, an additional publication by Otsuka et al. [16] was identified. After the elimination of 238 duplicate entries from the dataset, a remaining set of 297 distinct and nonredundant research papers was obtained, of which 9 were not accessible with our institutional login, and 1 was not written in English. This resulted in a total of 287 eligible records. Further analysis of the remaining publications revealed a substantial portion of papers irrelevant to the specific research objectives. Consequently, these nonrelevant papers were effectively filtered out from the final selection. The filtration process primarily centered around papers lacking defined features, particularly those that used machine learning models for blood pressure estimation based on extracted features or publications that did not extract time-dependent PPG features (*n* = 187). The second most prevalent reason for the exclusion of papers from the final selection was that a relationship between PPG features and blood pressure was not established in these studies (*n* = 71). Thirteen papers defined the features used but did not report the significance of a specific feature. After the rigorous filtering process based on undefined features, lack of relevance to blood pressure and PPG features, and other exclusion criteria, a total of 271 publications were excluded from the final selection, which finally led to the inclusion of 16 papers in this study. Figure 1 summarizes the results of the literature review.

In the following sections, the most significant findings of the literature search are discussed. The analysis will be centered on the examination of the most critical columns in Table 1, which represent the key findings from the selected publications. We tried to be as specific as possible, but because the publications were not uniform in the notations, we used the term “N/R”, denoting “not reported”, where the precise definition was not given in a study.

### 3.1. Publication Year and Measurement Site

The broad year range spanning from 1998 to 2023 observed in the retrieved publications indicates that the significance of specific PPG features in relation to blood pressure has been a topic of enduring interest over time. As anticipated, the finger, particularly the left index finger, appears to be the most commonly utilized measurement site in the assessed publications. The choice of the finger as the measurement site can be ascribed to its accessibility and ease of application. Furthermore, the index finger is less susceptible to motion artifacts, which leads to reliable PPG signals.

### 3.2. Number of Participants and Ages

The total number of participants in a study is indicated as “*n*”. In instances where hypertensive, normotensive, or hypotensive patients were included and a detailed number regarding the distribution of participants based on their blood pressure status is given, the specifics are also recorded. We indicated cases where patients with different blood pressure ranges were involved but the specific number was not defined as N/R. In papers where only the variable “*n*” was reported without additional context, it is not clear if the study included hypertensive and hypotensive subjects. The sample size ranged from a minimum of *n* = 5 to a maximum of *n* = 4374 individuals. This wide-ranging distribution of participant numbers reflects the diversity and variability in sample sizes across the reviewed publications.

The age distribution in most studies was presented as the mean ± standard deviation. These studies predominantly examined adults and elderly individuals. One publication by Liang et al. [17] did not report the age of the study population. Tendentiously, the age of the hypertensive groups was higher than that of the normotensive groups. This tendency aligns with the medical knowledge that identifies age as a risk factor of hypertension.

### 3.3. Environment and Measurement Time

Most studies were conducted in a clinical environment, which is indicative of the controlled and standardized conditions under which the research was undertaken. Moreover, in certain publications, details regarding the room temperature during data collection were explicitly outlined. Only one study was not conducted in a clinical environment but rather in a company during a medical checkup [18].The measurement times ranged from a few seconds to 10 min. The study by Liang et al. involved the extraction and analysis of three waveform measurements, each lasting 2.1 s [19]. Conversely, in the study by Otsuka et al. [18], multiple waveforms, each of a 5 s’ duration, were collected and averaged. The study by Feng et al. [20] included the acquisition of three separate measurements for every subject, each lasting 5 min, which were averaged for analysis. In the study, Liang et al. [17] collected 120 PPG signals, each lasting 1 s, for each patient, and they effectively collected 120-s PPG signals for each participant. Meanwhile, Chen et al. [21] used a collection methodology involving two to three instances of 1-min-long signals for each individual. In the residual publications, the signals with the specified measurement time were collected. Afterward, features were extracted from the acquired signals.

### 3.4. Systolic and Diastolic Blood Pressures

In this section, systolic (SBP) and diastolic blood pressures (DBP) are defined in millimeters of mercury (mmHg). Three studies by Kohjitani et al. [22], Tabara et al. [23], and Otsuka et al. [16,18] defined the mean value but did not specify the blood pressure of the hypertensive and normotensive groups. The mean blood pressure in the study by Otsuka et al. [18] was within the normotensive range, which can be explained by the higher proportion of normotensive patients than hypertensive patients in the study. Similarly, in the study by Kohjitani et al. [22], where the number of normotensive patients was not clearly defined, we can infer that the normotensive group outweighed the hypertensive group, resulting in the reported healthy blood pressure. The nearly equal proportion of hypertensive and normotensive patients in the publication by Tabara et al. [23] contributes to the slightly increased average blood pressure reported in the study. The elevated blood pressure in the study by Otsuka et al. [16] can be explained by the same principle, even though specific numbers of hypertensive and normotensive subjects were not provided. The study by Feng et al. [20] analyzed PPG waveforms obtained from pregnant women with and without preeclampsia. Thus, it is not surprising that the blood pressures in the healthy group were lower than those in the preeclampsia group. In three studies by Liang et al. [19], Echeverría et al. [24], and Chen et al. [21], the blood pressure values were not explicitly reported; instead, the ranges corresponding to the different blood pressure classifications were defined. Two studies did not define the blood pressure [17,25]. In summary, while detailed classification data may not have been clarified, in the studies analyzed, careful interpretations of patient proportions in each group and blood pressure can be made.

### 3.5. Population Comparison

The calculated *p* value is an optimal indicator of the statistical significance of specific features in relation to blood pressure. To calculate the *p* values in six analyzed studies [18,20,21,22,26,27], different PPG time-dependent features were extracted from hypertensive and normotensive patients and compared. Furthermore, in the study by Liang et al. [17], data from prehypertensive subjects were analyzed. This additional focus on prehypertension provides valuable insights into the potential early markers of blood pressure changes. The publication by Echeverría et al. [24] is the only reviewed paper that evaluated data from hypotensive patients and compared these to hypertensive and normotensive datasets. In some publications [16,17,23,28,29], the significance of the correlation of blood pressure and PPG signals was investigated. The study by Takazawa et al. [25] focused on the relationships between PPG features before and after the administration of vasoactive drugs. These drugs, namely angiotensin and nitroglycerin, increase blood pressure via vasoconstriction and decrease blood pressure via vasodilation. In contrast to other studies, one study by Jeong et al. [30] did not compare PPG data from groups with different blood pressures. Instead, a novel perspective was used by analyzing data collected before and after two levels of cycling exercises. Zhang et al. [31] compared PPG signals from untreated hypertensive subjects with PPG data from healthy subjects. First, they calculated the *p* value between the two groups before conducting a cardiopulmonary exercise test (CPET). An additional comparison was conducted subsequent to the completion of the CPET by both groups. Finally, as already mentioned, a study by Feng et al. [20] compared PPG signals from a preeclampsia group with those from a non-preeclampsia group.

### 3.6. SBP and DBP Results

The predominantly reported feature of SBP, which appeared 14 times, is the *d* wave-to-*a* wave ratio (d/a), followed by the *b* wave-to-*a* wave (b/a) with seven counts and (b−c−d)/a with six counts. Moreover, (b−c−d−e)/a, ${\left(S_{+1}+c_{-1}\right)}^{2}/{\left(O_{+1}+O_{+1}^{\prime }\right)}^{2}$, $(b_{-2}-d_{-2})/bd$, $Sc_{-2}$, $c_{-2}/S$, $c_{-1}/w$, and $\left(S-c_{-2}\right)/Sc_{-2}$ were reported four times, and the time span from the onset to the systolic peak (AT), $Sd_{-2}$, *d*, and the systolic amplitude (*S*) was reported three times. The ratios of *c* wave to *a* wave (c/a) and *e* wave to *a* wave (e/a) were both identified as significant features twice. Several features, while described only once, exhibited distinct characteristics or calculations, such as ${\left(S_{+1}+c_{-1}\right)}^{2}$, (b−e)/a, (c+d−b)/a, $b_{-2}/S$, and ${\left(S_{+1}+w\right)}^{2}/{\left(O_{+1}+O_{+1}^{\prime }\right)}^{2}$. Other better-known features such as *t*, db, DT, −d/a, and (b−d−e)/a were reported only once. In the study by Park et al. [29], the *p* values for the (b−c−d)/a and d/a ratios were calculated using age as a covariant. The area difference ratio (ADR) is a novel feature, introduced by Feng et al. [20], and it describes the difference between two areas: the area of the triangle defined by the points *S*, *O*, and $O^{\prime }$, and the area under the curve, traced by these same points. Another interesting feature was suggested by Echeverría et al. [24], where they used the position of the dicrotic notch (*D*) in relation to the systolic peak as an indicator of blood pressure.

**Figure 2:**
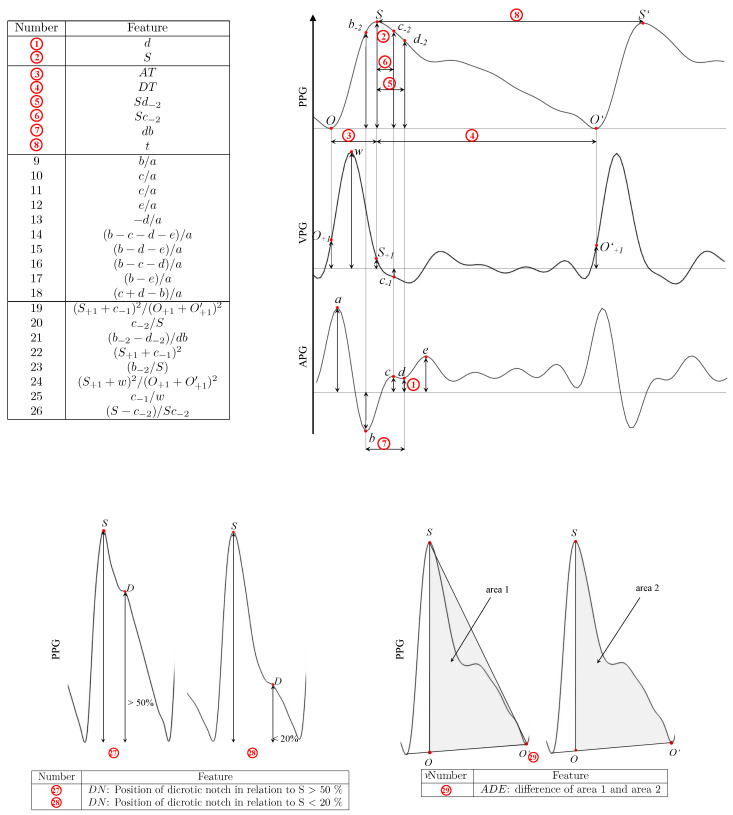
Visual representation of the 29 features extracted from PPG, VPG, and APG waveforms that correlate with blood pressure. The features include amplitude-based and time-based measurements, such as the dicrotic notch, systolic peak, and augmentation time. The detailed descriptions of each feature are provided in the main text. Abbreviations: PPG: photoplethysmogram, VPG: velocity photoplethysmogram, APG: acceleration photoplethysmogram.

**Figure 3:**
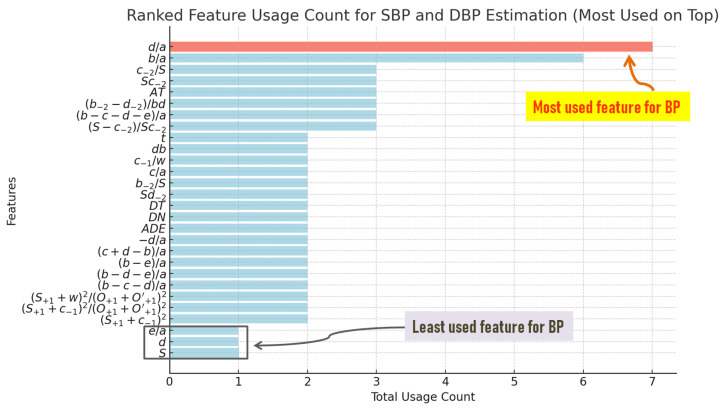
Frequency of features used in systolic and diastolic blood pressure estimation. The most frequently used feature, d/a, is highlighted in red at the top, appearing in 7 studies, followed by b/a with 6 counts. Other features, such as (b−c−d−e)/a, $(b_{-2}-d_{-2})/bd$, and $\left(S-c_{-2}\right)/Sc_{-2}$, were each used in 3 studies. The least used features, including *S*, *d*, e/a, and $c_{-1}/w$, are grouped and highlighted in a blue box at the bottom of the chart.

Most of the reviewed studies focused on investigating features in relation to SBP. In contrast, the evaluation of features connected with DBP was relatively less prevalent in these studies. However, analogous to the findings for SBP, the *d/a* ratio also appeared as the most frequently reported feature for DBP, with 6 instances, followed by the *b/a* ratio, which was mentioned twice. Other features, such as (b−c−d−e)/a and *db*, were only mentioned once. Figure 2 explains the features, and Figure 3 summarizes the results for SBP in a bar chart.

**Table 1:** Overview of the reviewed papers. Abbreviations: SBP: systolic blood pressure, DBP: diastolic blood pressure, BP: blood pressure, PE: preeclampsia, NPE: non preeclampsia, APG: acceleration photoplethysmogram, N/R: not reported.

Publication Year	Measurement Site	Number of Participants	Age	Environment	Measurement Time	SBP (mmHg)	DBP (mmHg)	Population Comparison	Optimal Feature SBP (Significance)	Optimal Feature DBP (Significance)	Reference
2023	Index finger	*n* = 26	62 ± 16	Clinical	1–10 min	Hypertension (SBP > 140 mmHg), normotension (SBP = 90 mmHg–140 mmHg) and hypotension (SBP < 90 mmHg)	N/R	Hypertension vs normotension and hypotension vs. normotension (during surgery)	Hypertension vs. normotension: *S* (*p* < 0.0001), dicrotic notch placed > 50% of *S* (*p* < 0.0001), hypotension vs. normotension: *S* (*p* = 0.021), dicrotic notch < 20% of *S* (*p* < 0.0001)	N/R	[24]
2021	N/R	*n* = 128 (normotensive: 45 prehypertensive: 45 hypertensive: 38)	Normotensive: 44.5 ± 16.3, hypertension: 61.6 ± 13.2	Clinical	2–3 s	Normotensive: 105.9 ± 8.9, hypertensive: 139.5 ± 15.4	Normotensive: 64.5 ± 7.3, hypertensive 76.3 ± 10.6	Hypertensive vs. normotensive	*S* (*p* = 0.016), AT (*p* = 0.002), $c_{-1}/w$ (*p* = 5.02 × 10^−4^), b/a (*p* = 1.77 × 10^−4^), d/a (*p* = 7.71 × 10^−8^), (b−c−d−e)/a (*p* = 1.40 × 10^−5^), (b−e)/a (*p* = 0.031), (b−c−d)/a (*p* = 3.11 × 10^−6^), (c+d−b)/a (*p* = 3.11 × 10^−6^), −d/a (*p* = 7.71 × 10^−8^)	N/R	[27]
2019	Index finger of left hand	*n* = 262	38.57 ± 11.64	Clinical	90 s	116.35 ± 12.49	71.24 ± 8.23	Correlation of BP and APG features	(Age as a covariant) d/a (*p* = 0.001), (b−c−d)/a (*p* = 0.041)	(Age as a covariant) d/a (*p* < 0.001)	[29]
2019	Index finger of left hand	*n* = 124 (hypertensive: 35, prehypertensive: 41, normotensive: 48)	55 ± 16 (hypertensive, normotensive and prehypertensive N/R)	Clinical	2.1 s	Hypertensive (SBP ≥140 mmHg or DBP ≥90 mmHg), prehypertensive group (SBP: 120–140 mmHg or DBP: 80–90 mmHg), and normotensive group (SBP < 120 and DBP < 80)	N/R	Correlation between BP and APG features	(b−c−d)/a (*p* = 0.0001), $(b_{-2}-d_{-2)}/db$ (*p* = 0.0009), $\left(S-c_{-2}\right)/Sc_{-2}$ (*p* = 0.0013), AT (*p* = 0.0017), $c_{-2}/S$ (*p* = 0.0425), $Sc_{-2}$ (*p* = 0.00003), ${\left(S_{+1}+c_{-1}\right)}^{2}/{\left(O_{+1}+O_{+1}^{\prime }\right)}^{2}$ (*p* = 0.0058), ($S_{+1}+c_{-1}$)^2^ (*p* = 0.0443), $b_{-2}/S$ (*p* = 0.0004), ${\left(S_{+1}+w\right)}^{2}/{\left(O_{+1}+O_{+1}^{\prime }\right)}^{2}$ (*p* = 0.0079)	N/R	[19]
2018	Finger	*n* = 30 (15 untreated hypertensive subjects, 15 normotensive subjects)	Hypertensive: 48 ± 11.5, healthy: 44 ± 11.8	Clinical, between 20 and 22 °C	N/R	Hypertensive: 142.8 ± 7.5, healthy: 112.8 ± 6.5	Hypertensive: 90.2 ± 11.0, healthy: 78.2 ± 11.3	Hypertensive vs. healthy, hypertensive after CPET vs. healthy after CPET	Before CPET: e/a (*p* = 0.007), after CPET: b/a (*p* = 0.03), (b−c−d−e)/a (*p* = 0.011), d/a (*p* = 0.024), c/a (*p* = 0.004)	N/R	[31]
2018	Index finger of left hand	*n* = 121 (normtension: 46, prehypertension: 41, hypertension: 34)	N/R	Clinical	120 s	N/R	N/R	Hypertensive vs. normotensive, normotensive vs. prehypertension, prehypertension vs. hypertension	*p* < 0.001: ${\left(S_{+1}+c_{-1}\right)}^{2}/{\left(O_{+1}+O_{+1}^{\prime }\right)}^{2}$, $(b_{-2}-d_{-2})/db$, $Sc_{-2}$, $c_{-2}/S$, $Sd_{-2}$, (b−c−d)/a, *d*, $c_{-1}/w$, d/a, $\left(S-c_{-2}\right)/Sc_{-2}$	N/R	[17]
2018	Index finger	*n* = 72 (36 PE, 36 healthy pregnant women)	NPE: 30.0 ± 3.6, PE: 31.3 ± 3.9	Clinical, 24 °C	5 min	NPE: 115.8 ± 9.2, PE: 152.6 ± 14.8	NPE: 69.6 ± 7.6, PE: 99.3±3.0	PE vs. NPE	ADE (*p* < 0.01)	N/R	[20]
2017	Index finger of right hand	*n* = 4373	68.1 ± 6.6	Clinical, 24 ± 26 °C	N/R	130.8 ± 17.2	75.6 ± 9.9	Correlation between BP and APG features	d/a (*p* < 0.001)	d/a (*p* < 0.001)	[28]
2016	Wrist	*n* = 30 (10 normotensive 20 hypertensive)	35 to 73	Clinical, 20 °C	1 min	Normotension (SBP < 140 mmHg)	Normotension (DBP < 90 mmHg)	Hypertensive vs. normotensive	DT (*p* = 0.001), AT (*p* = 0.025), *t* (*p* = 0.001)	N/R	[21]
2016	Finger	*n* = 1613 (hypertension: 829, normotension: 784)	65.3 ± 9.6 (normotensive and hypertensive N/R)	Clinical	20 s	134 ± 19 (normotensive and hypertensive N/R)	76 ± 11 (normotensive and hypertensive N/R)	Correlation between BP and APG features	b/a (*p* < 0.001), d/a (*p* < 0.001), (b−c−d−e)/a (*p* < 0.001), (b−d−e)/a (*p* < 0.001)	N/R	[23]
2014	Index finger of left hand	*n* = 168 (hypertensive: 41, normotensive: N/R)	44.4 ± 18.8 (normotensive and hypertensive N/R)	Clinical	90 s	115.2 ± 15.3 (hypertension and normotension N/R)	69.8 ± 12.0 (hypertension and normotension N/R)	Hypertensive (24.4% of participants) vs. normotensive	b/a (*p* = 0.0412), c/a (*p* = 0.0110), d/a (*p* < 0.001)	d/a (*p* < 0.0001)	[22]
2013	Index finger	*n* = 5	26 to 50	Clinical	30 s	127.67 ± 17.063	71.04 ± 7.33	Comparison before and after 2 levels of cycling exercises	d/a (*p* < 0.01), e/a (*p* < 0.01), db (*p* < 0.01)	db (*p* < 0.01)	[30]
2007	Index finger of left hand	*n* = 973 (hypertensive: 110, normotension: 863)	44 ± 6 (normotensive and hypertensive N/R)	Medical checkup at a company 22 ± 2 °C	5 s	119 ± 13 (normotensive and hypertensive N/R)	76 ± 10 (normotensive and hypertensive N/R)	Normotensive vs hypertensive	b/a (*p* = 0.003), d/a (*p* < 0.001)	b/a (*p* = 0.004), d/a (*p* < 0.001)	[18]
2006	Index finger of left hand	*n* = 211 (normotensive and hypertensive N/R)	63 ± 15 (normotensive and hypertensive N/R)	Clinical	N/R	130 ± 18 (normotensive and hypertensive N/R)	76 ± 12 (normotensive and hypertensive N/R)	Correlation between BP and APG features	b/a (*p* < 0.001), d/a (*p* < 0.001)	d/a (*p* < 0.01)	[16]
2005	Index finger of left hand	*n* = 848 (normotensive: 544, hypertensive: 304)	Normotensive: 59.0 ± 11.8, hypertensive: 63.9 ± 9.3	Clinical	N/R	Normotensive: 122.5 ± 11.1, hypertensive: 154.1 ± 13.8	Normotensive: 69.5 ± 8.5, hypertensive: 83.3 ± 10.5	544 normotensive vs. 304 hypertenisive	b/a (*p* < 0.001), d/a (*p* < 0.001), (b−c−d−e)/a (*p* < 0.001)	b/a (*p* < 0.001), d/a (*p* < 0.001), (b−c−d−e)/a (*p* < 0.001)	[26]
1998	Index finger of left hand	*n* = 39	54 ± 11	Clinical	N/R	N/R	N/R	Before/after vasoactive agents applied	d/a (*p* < 0.01)	N/R	[25]

### 3.7. Feature Descriptions

In this study, 29 distinct features were extracted from PPG, VPG, and APG signals for their correlation with blood pressure. The following are the key features analyzed:

- **d/a**: Ratio of the dicrotic notch (*d*) amplitude to the systolic peak (*a*).
- **b/a**: Ratio of the first inflection point (*b*) to the systolic peak (*a*).
- **(b−c−d)/a**: Combined difference of the *b*, *c*, and *d* amplitudes, normalized by the systolic peak (*a*).
- **(b−c−d−e)/a**: Combined difference of the *b*, *c*, *d*, and *e* amplitudes, normalized by the systolic peak (*a*).
- **${\left(S_{+1}+c_{-1}\right)}^{2}/{\left(O_{+1}+O_{+1}^{\prime }\right)}^{2}$**: Complex ratio involving the sum of peaks and baseline oscillations.
- **$(b_{-2}-d_{-2})/bd$**: Difference between secondary inflections, normalized by the systolic and diastolic time difference.
- **$Sc_{-2}$**: Amplitude of the second derivative of the systolic peak.
- **$c_{-2}/S$**: Ratio of the secondary inflection of the systolic peak to the systolic amplitude.
- **$c_{-1}/w$**: Ratio of the first inflection amplitude to the waveform width.
- **$\left(S-c_{-2}\right)/Sc_{-2}$**: Difference between the systolic peak and the second derivative of the inflection point, normalized by the second derivative amplitude.
- **Augmentation Time (AT)**: Time from the onset of the PPG waveform to the systolic peak.
- **Deceleration Time (DT)**: Time from the systolic peak to the end of the dicrotic notch.
- **db**: Time between the dicrotic notch and the minimum amplitude in the APG waveform.
- ***t***: Total time duration of the PPG waveform.
- **Area Difference Ratio (ADR)**: A novel feature introduced by Feng et al. representing the difference between two areas formed by the PPG waveform.
- **Dicrotic Notch Position (DN)**: Location of the dicrotic notch relative to the systolic peak.
- **(b−e)/a**: Ratio between the *b* and *e* amplitudes, normalized by the systolic peak (*a*).
- **(c+d−b)/a**: Difference between *c*, *d*, and *b*, normalized by the systolic peak (*a*).
- **${\left(S_{+1}+c_{-1}\right)}^{2}$**: Square of the sum of $S_{+1}$ and $c_{-1}$.
- **$b_{-2}/S$**: Ratio of $b_{-2}$ to the systolic peak (*S*).
- **${\left(S+w\right)}^{2}/{\left(O_{+1}+O_{+1}^{\prime }\right)}^{2}$**: Complex ratio involving *S* and baseline oscillations.
- **$\left(S-c_{-2}\right)/Sc_{-2}$**: Difference between the systolic peak and $c_{-2}$, normalized by the second derivative of the systolic peak.
- **DN(S>50%)**: Marks the position of the dicrotic notch relative to the systolic peak (*S*) when *S* is greater than 50%.
- **DN(S<20%)**: Marks the position of the dicrotic notch when *S* is less than 20%.

These features provide insight into blood pressure fluctuations by leveraging time-based and amplitude-based characteristics of the waveform. Detailed information on the naming conventions for most of the features discussed can be found in [32]. Figure 2 illustrates these features visually, summarizing their clinical relevance and potential for blood pressure estimation.

Figure 3 illustrates the frequency with which various features were used in studies related to systolic blood pressure. The most reported feature is the d/a ratio, appearing in seven studies, followed by the b/a ratio with six counts. Other features, such as (b−c−d−e)/a, $(b_{-2}-d_{-2})/bd$, and $\left(S-c_{-2}\right)/Sc_{-2}$, were each used in three studies. In contrast, several features, including *S*, *d*, e/a, and $c_{-1}/w$, were only utilized once. These least used features are grouped and highlighted in a blue box at the bottom of the chart, while the most used feature, d/a, is emphasized at the top in red.

## 4. Discussion

The ratios d/a and b/a are clearly the most-reviewed significant features, whereas the other ratios in the second derivative (c/a and e/a) appeared to be not as important in relation to blood pressure staging. Indeed, c/a and e/a were mentioned as not significant six and five times, respectively. This leads to the conclusion that these features are quite well investigated, whereas d/a and b/a play significant roles in blood pressure. The observed significance of the ratios can be attributed to their physiological relevance. The *a* and *b* waves reflect early systolic ejection, a phenomenon linked to arterial stiffness and compliance, both of which play an important role in influencing blood pressure dynamics. Moreover, research has described a negative correlation between the |b/a| ratio and age, further highlighting the potential of the |b/a| ratio as an informative marker [33]. On the other hand, the *d* wave corresponds to the late systolic phase, while the d/a ratio provides information about vascular resistance and correlates with blood augmentation [16,26]. The physiological meaning of the c/a and e/a ratios is still not very well understood [22]. The (b−c−d)/a and (b−c−d−e)/a are both markers of vascular aging and, unsurprisingly, correlate with blood pressure [26,29].

Blood pressure estimation via PPG signals has seen a proliferation of features contributing to accurate readings. While established features are often the focal point of research, several developing ones, such as AT, have garnered attention, albeit in limited studies. Their precise roles and significance in relation to blood pressure remain a topic of exploration and could unlock pivotal insights for the field.

Modern medicine is leaning heavily into the capabilities of machine learning, with a specific emphasis on its potential to gauge blood pressure via PPG signals. A common concern, however, is that many machine learning methodologies tend to operate as ‘black boxes’, making them opaque and challenging to interpret in a medical context. In contrast, PPG parameters, grounded in human physiology, offer tangible, explainable results. This study aims to bridge the knowledge gap, emphasizing the significance of particular features and their correlation with blood pressure.

Our review encompasses 29 features, yet the field of PPG signal analysis offers more. Various studies introduce features, often embedded in machine learning models for blood pressure estimation, without explicit definitions or significance testing. For instance, several studies highlight pulse arrival time computation, necessitating both PPG and electrocardiogram signals [34,35,36]. Given our focus on PPG signals exclusively, electrocardiography was outside our purview. This disparity underscores the existence of a plethora of PPG features awaiting systematic examination concerning blood pressure. Additionally, the inconsistent feature descriptions across studies pose challenges in comparative analysis and interpretation.

Solà et al. [37] made a notable contribution, demonstrating the feasibility of blood pressure estimation using pulse transit time measurements derived from chest-mounted sensors. However, it is crucial to address some exclusions, such as the study by Iketani et al. [38]. While it delved into the interplay between blood pressure and PPG features, its focus on a younger, healthy demographic with non-significant outcomes made it less relevant for our present review.

This review underscores several novel findings in the context of PPG-based blood pressure estimation. Most notably, the identification of underutilized temporal domain features, such as (b−c−d)/a and (b−c−d−e)/a, ${\left(S_{+1}+c_{-1}\right)}^{2}/{\left(O_{+1}+O_{+1}^{\prime }\right)}^{2}$ demonstrates their potential in improving classification accuracy for blood pressure levels. Additionally, our comprehensive analysis reveals that features derived from the second derivative of PPG, such as the d/a and b/a ratios, remain the most significant in the current literature. These findings could pave the way for future investigations aiming to standardize feature selection in machine learning models for blood pressure monitoring.

It is important to recognize that while the b/a and d/a ratios are well established, novel features such as the ADR offer promising opportunities for further research. Our review emphasizes the need for standardized definitions of PPG features across studies to enable better comparisons and interpretation.

One limitation in PPG-based blood pressure estimation is the accuracy of feature selection, which often requires calibration with established techniques, such as sphygmomanometers, to ensure reliability. In line with recent recommendations [39], it is crucial to standardize feature selection and evaluation criteria to enhance accuracy. This is particularly relevant for novel features like ADR, which require robust calibration to ensure clinical reliability. Addressing limitations in current datasets, including demographic diversity, is essential for improving model generalizability.

Future studies should prioritize validating PPG-based methods through comparison with mature techniques to enhance their accuracy and clinical applicability. Additionally, the process of feature calibration warrants further investigation.

Despite advancements, PPG-based blood pressure estimation still faces challenges. Current studies often lack validation across diverse populations, and many machine learning models remain ’black boxes,’ limiting their clinical interpretability. Future research should focus on refining feature extraction techniques, validating models in larger, more diverse cohorts, and developing explainable AI tools that align with clinical needs.

## 5. Conclusions

Among the 29 analyzed features, the b/a and d/a ratios were the most frequently reported as significant for blood pressure estimation. Features derived from the second derivative, involving the *a*, *b*, *c*, *d*, and *e* waves, show strong potential for estimating blood pressure across different ranges. Although promising new features emerged, they were excluded due to insufficient statistical validation. Most studies reviewed focused on hypertensive and normotensive ranges, highlighting the need for research on prehypertensive and hypotensive ranges. While PPG features are increasingly used for blood pressure interpretation, further investigation into the physiological mechanisms behind them is needed. Our findings support the potential for PPG signals in cuffless, continuous blood pressure monitoring.

## Data Availability

The authors declare that all data supporting the findings of this study are available within the paper.

## References

[1] World Health Organization Fact Sheets-Hypertension. https://www.who.int/news-room/fact-sheets/detail/hypertension.

[2] Ji J., Dong M., Lin Q., Tan K.C. (2023). Noninvasive cuffless blood pressure estimation with dendritic neural regression. IEEE Trans. Cybern..

[3] Centers for Disease Control and Prevention Facts about Hypertension. https://www.cdc.gov/bloodpressure/facts.htm.

[4] Gupta S., Singh A., Sharma A. (2022). Dynamic large artery stiffness index for cuffless blood pressure estimation. IEEE Sens. Lett..

[5] Yao P., Xue N., Yin S., You C., Guo Y., Shi Y. (2022). Multi-dimensional feature combination method for continuous blood pressure measurement based on wrist ppg sensor. IEEE J. Biomed. Health Inform..

[6] Ali N.F., Atef M. (2022). Lstm multi-stage transfer learning for blood pressure estimation using photoplethysmography. Electronics.

[7] El Hajj C., Kyriacou P.A. Recurrent neural network models for blood pressure monitoring using ppg morphological features. Proceedings of the 2021 43rd Annual International Conference of the IEEE Engineering in Medicine and Biology Society (EMBC).

[8] Chen J.-W., Huang H.-K., Fang Y.-T., Lin Y.-T., Li S.-Z., Chen B.-W., Lo Y.-C., Chen P.-C., Wang C.-F., Chen Y.-Y. (2022). A data-driven model with feedback calibration embedded blood pressure estimator using reflective photoplethysmography. Sensors.

[9] Maqsood S., Xu S., Springer M., Mohawesh R. (2021). A benchmark study of machine learning for analysis of signal feature extraction techniques for blood pressure estimation using photoplethysmography (ppg). IEEE Access.

[10] Elgendi M. (2012). On the analysis of fingertip photoplethysmogram signals. Curr. Cardiol. Rev..

[11] Allen J. (2007). Photoplethysmography and its application in clinical physiological measurement. Physiol. Meas..

[12] Elgendi M. (2020). PPG Signal Analysis: An Introduction Using MATLAB^®^.

[13] Elgendi M., Fletcher R., Liang Y., Howard N., Lovell N.H., Abbott D., Lim K. (2019). The use of photoplethysmography for assessing hypertension. NPJ Digit. Med..

[14] Chowdhury M.H., Shuzan M.N.I., Chowdhury M.E.H., Mahbub Z.B., Uddin M.M., Khandakar A., Reaz M.B.I. (2020). Estimating blood pressure from the photoplethysmogram signal and demographic features using machine learning techniques. Sensors.

[15] Duan K., Qian Z., Atef M., Wang G. A feature exploration methodology for learning based cuffless blood pressure measurement using photoplethysmography. Proceedings of the 2016 38th Annual International Conference of the IEEE Engineering in Medicine and Biology Society (EMBC).

[16] Otsuka T., Kawada T., Katsumata M., Ibuki C. (2006). Utility of second derivative of the finger photoplethysmogram for the estimation of the risk of coronary heart disease in the general population. Circ. J..

[17] Liang Y., Chen Z., Ward R., Elgendi M. (2018). Hypertension assessment via ecg and ppg signals: An evaluation using mimic database. Diagnostics.

[18] Otsuka T., Kawada T., Katsumata M., Ibuki C., Kusama Y. (2007). Independent determinants of second derivative of the finger photoplethysmogram among various cardiovascular risk factors in middle-aged men. Hypertens. Res..

[19] Liang Y., Chen Z., Ward R., Elgendi M. (2019). Hypertension assessment using photoplethysmography: A risk stratification approach. J. Clin. Med..

[20] Feng Y., Drzymalski D., Zhao B., Wang X., Chen X. (2018). Measurement of area difference ratio of photoplethysmographic pulse wave in patients with pre-eclampsia. BMC Pregnancy Childbirth.

[21] Chen Y., Zhu Y., Ma H.T., Huang H. A study of photoplethysmography intensity ratio in hypertension. Proceedings of the 2016 IEEE International Conference on Real-time Computing and Robotics (RCAR).

[22] Kohjitani A., Miyata M., Iwase Y., Ohno S., Tohya A., Manabe Y., Hashiguchi T., Sugiyama K. (2014). Associations between the autonomic nervous system and the second derivative of the finger photoplethysmogram indices. J. Atheroscler. Thromb..

[23] Tabara Y., Igase M., Okada Y., Nagai T., Miki T., Ohyagi Y., Matsuda F. (2016). Usefulness of the second derivative of the finger photoplethysmogram for assessment of end-organ damage: The j-shipp study. Hypertens. Res..

[24] Echeverría N.I., Scandurra A.G., Acosta C.M., Meschino G.J., Sipmann F.S., Tusman G. (2023). Photoplethysmography waveform analysis for classification of vascular tone and arterial blood pressure: Study based on neural networks. Rev. Esp. Anestesiol. Reanim. (Engl. Ed.).

[25] Takazawa K., Tanaka N., Fujita M., Matsuoka O., Saiki T., Aikawa M., Tamura S., Ibukiyama C. (1998). Assessment of vasoactive agents and vascular aging by the second derivative of photoplethysmogram waveform. Hypertension.

[26] Hashimoto J., Watabe D., Kimura A., Takahashi H., Ohkubo T., Totsune K., Imai Y. (2005). Determinants of the second derivative of the finger photoplethysmogram and brachial-ankle pulse-wave velocity: The Ohasama study. Am. J. Hypertens..

[27] Yao L.-P., Liu W.-Z. (2021). Hypertension assessment based on feature extraction using a photoplethysmography signal and its derivatives. Physiol. Meas..

[28] Inoue N., Kawakami H., Yamamoto H., Ito C., Fujiwara S., Sasaki H., Kihara Y. (2017). Second derivative of the finger photoplethysmogram and cardiovascular mortality in middle-aged and elderly japanese women. Hypertens. Res..

[29] Park Y.-J., Lee J.-M., Kwon S.-H. (2019). Association of the second derivative of photoplethysmogram with age, hemodynamic, autonomic, adiposity, and emotional factors. Medicine.

[30] Jeong I., Finkelstein J. Applicability of the second derivative photoplethysmogram for non-invasive blood pressure estimation during exercise. Proceedings of the 2013 Pan American Health Care Exchanges (PAHCE).

[31] Zhang Y., Jiang Z., Qi L., Xu L., Sun X., Chu X., Liu Y., Zhang T., Greenwald S.E. (2018). Evaluation of cardiorespiratory function during cardiopulmonary exercise testing in untreated hypertensive and healthy subjects. Front. Physiol..

[32] Elgendi M., Liang Y., Ward R. (2018). Toward generating more diagnostic features from photoplethysmogram waveforms. Diseases.

[33] Imanaga I., Hara H., Koyanagi S., Tanaka K. (1998). Correlation between wave components of the second derivative of plethysmogram and arterial distensibility. Jpn. Heart J..

[34] Freithaler M., Chandrasekhar A., Dhamotharan V., Landry C., Shroff S.G., Mukkamala R. (2023). Smartphone-based blood pressure monitoring via the oscillometric finger pressing method: Analysis of oscillation width variations can improve diastolic pressure computation. IEEE Trans. Biomed. Eng..

[35] Hsu Y.-C., Li Y.-H., Chang C.-C., Harfiya L.N. (2020). Generalized deep neural network model for cuffless blood pressure estimation with photoplethysmogram signal only. Sensors.

[36] Dave T., Pandya U., Joshi M. Cuff-less blood pressure measurement from wireless ecg and ppg signals. Proceedings of the 2021 IEEE International Symposium on Smart Electronic Systems (iSES).

[37] Solà J., Proença M., Ferrario D., Porchet J.-A., Falhi A., Grossenbacher O. (2013). Noninvasive and nonocclusive blood pressure estimation via a chest sensor. IEEE Trans. Biomed. Eng..

[38] Iketani Y., Iketani T., Takazawa K., Murata M. (2000). Second derivative of photoplethysmogram in children and young people. Jpn. Circ. J..

[39] Elgendi M., Haugg F., Fletcher R.R., Allen J., Shin H., Alian A., Menon C. (2024). Recommendations for evaluating photoplethysmography-based algorithms for blood pressure assessment. Commun. Med..

